# Spirituality and Religiosity in Adults With Head and Neck Cancer: A Scoping Review of Concepts, Measures, and Outcomes

**DOI:** 10.1111/odi.70335

**Published:** 2026-04-14

**Authors:** Liliana Wolf Braun, Caique Mariano Pedroso, Helena Miguel Cotter, David Dean, Ana Carolina Prado‐Ribeiro, Manoela Domingues Martins, Thomas P. Sollecito, Horace M. DeLisser, Alan Roger Santos‐Silva

**Affiliations:** ^1^ Oral Diagnosis Department Piracicaba Dental School, University of Campinas Piracicaba São Paulo Brazil; ^2^ Oral Medicine Service Fred Hutchinson Cancer Center Seattle Washington USA; ^3^ Department of Oral Medicine University of Washington Seattle Washington USA; ^4^ Instituto Do Câncer Do Estado de São Paulo Octavio Frias de Oliveira (ICESP) São Paulo Brazil; ^5^ Department of Oral Pathology, School of Dentistry Universidade Federal do Rio Grande do Sul Porto Alegre Rio Grande do Sul Brazil; ^6^ Department of Oral Medicine University of Pennsylvania School of Dental Medicine Philadelphia Pennsylvania USA; ^7^ Perelman School of Medicine. Pulmonary, Allergy and Critical Care Division, Department of Medicine University of Pennsylvania Philadelphia Pennsylvania USA

**Keywords:** head and neck cancer, holistic care, spiritual care, spirituality

## Abstract

**Objective:**

To synthesize evidence on spirituality in patients with head and neck cancer (HNC), focusing on its dimensions, assessment strategies, and impact on health outcomes.

**Methods:**

A comprehensive search of PubMed, Embase, LILACS, Web of Science, Scopus, and gray literature identified qualitative and quantitative studies explicitly addressing spirituality in HNC. Eligible sources were narratively synthesized.

**Results:**

Twenty‐one studies met inclusion criteria. Core spiritual dimensions included the search for meaning, inner peace, religious faith, and a positive outlook. Six studies reported associations between spirituality and improved quality of life (QoL), while three described enhanced coping capacity and emotional resilience. Religious faith, irrespective of denomination, was associated with better pain management, fewer treatment‐related side effects, lower existential distress, and, in some studies, improved survival outcomes. The Functional Assessment of Chronic Illness Therapy–Spiritual Well‐being Scale was the most frequently applied instrument, assessing beliefs, meaning, and inner peace.

**Conclusion:**

Spirituality was frequently associated with coping capacity, psychological adjustment, and QoL in patients with HNC. Integrating spiritual care into holistic oncology practice may enhance treatment adherence, resilience, and patient well‐being.

## 1 Introduction

Head and neck cancer (HNC) accounts for approximately 660,000 new cases and 325,000 deaths each year, making it the seventh most common malignancy worldwide (Hu et al. 2025). The head and neck area contains vital anatomical structures that are frequently invaded or compressed by malignant tumors, resulting in functional impairments such as dysphagia, dyspnea, speech difficulties, sensory loss, trismus, hyposalivation, and facial disfigurement (Hu et al. 2025; Li et al. 2022). In addition to these physical consequences, patients often experience significant psychosocial burdens, including depression, social withdrawal, negative body image, anxiety, and an increased risk of suicide (Hu et al. 2025; Chang et al. 2022). Despite this multidimensional impact, clinical management has traditionally emphasized disease‐free survival, frequently overlooking psychosocial and spiritual aspects of care (Musa et al. 2024).

Spirituality is increasingly recognized as a critical component of comprehensive healthcare, particularly for its role in fostering meaning, purpose, and inner coherence in individuals confronting serious illness (Whitford and Olver 2012; Ripamonti et al. 2016; Balboni et al. 2022) Spiritual well‐being is often understood not as adherence to specific religious doctrines, but as a broader experience of inner peace, meaning, and purpose (Whitford and Olver 2012). This dimension of well‐being may be expressed through religious beliefs and practices, emotional and social relationships, community engagement, or a sense of connection with nature, culture, or the arts. Such expressions have been linked to improved emotional well‐being, enhanced hope, and more adaptive coping strategies, all of which positively influence quality of life (QoL) during health adversity (Whitford and Olver 2012; Ripamonti et al. 2016). Nevertheless, between 49% and 91% of seriously ill patients do not report receiving spiritual care (Balboni et al. 2022).

For patients with HNC, the challenges are unique and include visible disfigurement, restrictions in daily activities, social isolation, and profound emotional distress resulting from the cancer itself and its treatment side effects (Goswami and Gupta 2019; Ferri et al. 2024). When facing these challenges, individuals may seek connections with peers who share the illness, consult spiritual advisors, process their concerns through writing or artistic expression, and turn to family members, friends, or faith communities for affirmation and support (Ferri et al. 2024). Higher levels of spirituality have been associated with greater emotional resilience, improved psychological adaptation, and reduced distress regarding changes in physical appearance, even in the presence of visible postsurgical sequelae (Becker et al. 2006; Reis et al. 2021).

Although spirituality has gained increasing recognition in the context of chronic illness, studies specifically examining its role among patients with HNC remain limited. This scoping review aims to synthesize the available evidence on spirituality in individuals diagnosed with HNC.

## 2 Methods

### 2.1 Protocol and Registration

This scoping review was conducted in accordance with the PRISMA‐ScR Checklist (Page et al. 2021). The guiding research question was: “What evidence is available regarding spirituality in patients with HNC?” The review protocol was registered on the Open Science Framework under registration number https://doi.org/10.17605/OSF.IO/87CAF.

### 2.2 Eligibility Criteria

Eligible studies included adult patients (≥ 18 years) diagnosed with HNC. Both quantitative and qualitative research designs were considered. To be included, studies had to explicitly address spirituality in this population. When spirituality was examined alongside other health outcomes, detailed spirituality‐specific data were required for inclusion. Religiosity was considered a dimension of spirituality, as defined in Section 2.10.

### 2.3 Population

HNC was defined as malignant tumors originating in the oral cavity, lips, oropharynx, hypopharynx, larynx, nasopharynx, nasal cavity, paranasal sinuses, salivary glands, vocal cords, or cervical lymph nodes. A small number of studies identified during the screening process included patient cohorts in which individuals with cutaneous basal cell carcinoma or esophageal squamous cell carcinoma were grouped with head and neck squamous cell carcinoma when examining spirituality and religiosity in oncology populations. These studies were retained because they were conducted within broader head and neck oncology care contexts and involved patients experiencing clinical and treatment‐related burdens comparable to those observed in HNC, including functional impairment, feeding difficulties, and potentially mutilating surgical procedures. This broader inclusion was adopted to capture oncologic conditions that may share similar psychosocial and functional burdens within the head and neck care context.

### 2.4 Setting

Studies conducted in any healthcare context, including hospitals, outpatient clinics, private practices, or home visits, were eligible. No geographic restrictions were applied.

### 2.5 Type of Evidence

Eligible sources included original research, systematic reviews, scoping and narrative reviews, observational studies, and case reports describing patient perceptions or spiritual interventions. Gray literature, such as dissertations, theses, clinical guidelines, institutional protocols, and healthcare reports, was also considered. Publications in English, Spanish, or Portuguese were eligible, without restrictions on publication date.

### 2.6 Exclusion Criteria

Studies were excluded if they: (1) did not specifically address HNC, basal cell skin carcinoma, or esophageal cancer; (2) had major methodological limitations or insufficient detail on spirituality; (3) were letters, opinions, editorials, conference abstracts, or posters; (4) lacked full texts or essential data; or (5) included participants younger than 18 years.

### 2.7 Information Sources and Search Strategy

A comprehensive search was conducted on December 16, 2024, across PubMed, Embase, LILACS, Web of Science, and Scopus. Gray literature was searched via ProQuest Dissertations & Theses and Google Scholar. The strategy combined keywords and controlled vocabulary (e.g., MeSH terms), including “head and neck cancer”, “spirituality”, “religiosity”, “spiritual care”, “spiritual well‐being”, “pastoral care”, “religious support”, “faith‐based care”, “spiritual counseling”, and “holistic care” (see Table S1 for the complete list). All references were imported into EndNote X9 (Thomson Reuters, Philadelphia, PA, USA) for automatic de‐duplication and manually verified.

### 2.8 Study Selection

After duplicate removal in EndNote X9, study selection proceeded in two phases. In Phase 1, two independent reviewers (L.W.B. and C.M.P.) screened titles and abstracts using Rayyan (Qatar Computing Research Institute). In Phase 2, the same reviewers evaluated full texts for eligibility. Disagreements were resolved through discussion or consulting with a third reviewer (A.R.S.‐S.).

### 2.9 Data Charting

Data extraction focused on population, concept, context, and key findings relevant to the review question. Variables included author, title, year, study design, sample size, participant age and sex, tumor site and stage, treatment type and stage, measures of spirituality and religiosity, coping strategies, instruments used to assess spirituality and QoL, and psychological outcomes. A preliminary extraction form was piloted and refined iteratively. Data were organized in Microsoft Excel Version 16 (Microsoft Corporation, Chicago, USA).

### 2.10 Data Items

Spirituality was conceptualized as a dynamic construct encompassing religious beliefs, as well as transcendent dimensions, such as meaning, purpose, and values. It may be expressed through social support, interpersonal relationships, connection with nature, engagement with the arts, and recognition of the sacred. Religiosity was considered a specific dimension of spirituality linked to individual beliefs and institutional practices. Spiritual needs included personal concerns, emotional and existential distress, and inner suffering, which are addressed through spiritual care provided by healthcare teams (Balboni et al. 2022). Sampling time points were categorized into different stages to reflect the patient trajectory. Spiritual care was defined as a respectful, patient‐centered response by healthcare professionals to the spiritual needs of individuals, encompassing sources of meaning, purpose, and transcendence, as well as addressing emotional, existential, and cultural concerns in a compassionate and integrated manner (Balboni et al. 2022).

### 2.11 Synthesis of Results

Findings were synthesized narratively, supported by descriptive statistics (e.g., frequencies and means) supported interpretation. Recurrent themes were categorized according to the conceptualization and measurement of spirituality, its impact on QoL, its role as a coping mechanism, and according to the four treatment stages (pretreatment, during treatment, posttreatment, and palliative care). The first stage encompassed the period immediately after diagnosis and prior to treatment, the second stage referred to the active treatment phase, the third stage involved immediate and follow‐up posttreatment assessment, and the fourth included assessment during palliative care. Studies that proposed professional approaches or identified key support needs were further described as professional care recommendations.

## 3 Results

### 3.1 Selection of Sources of Evidence

A total of 604 records were identified from electronic databases. After removing 268 duplicates, 336 records were screened by title and abstract, of which 254 were excluded. Eighty‐two full texts were assessed, with 15 meeting eligibility criteria. Additionally, 166 gray literature records were identified; after duplicate removal, 146 were screened, with 121 excluded. Twenty‐five full texts were assessed, and 6 were included. In total, 21 studies were incorporated into this scoping review (Figure 1).

**FIGURE 1:**
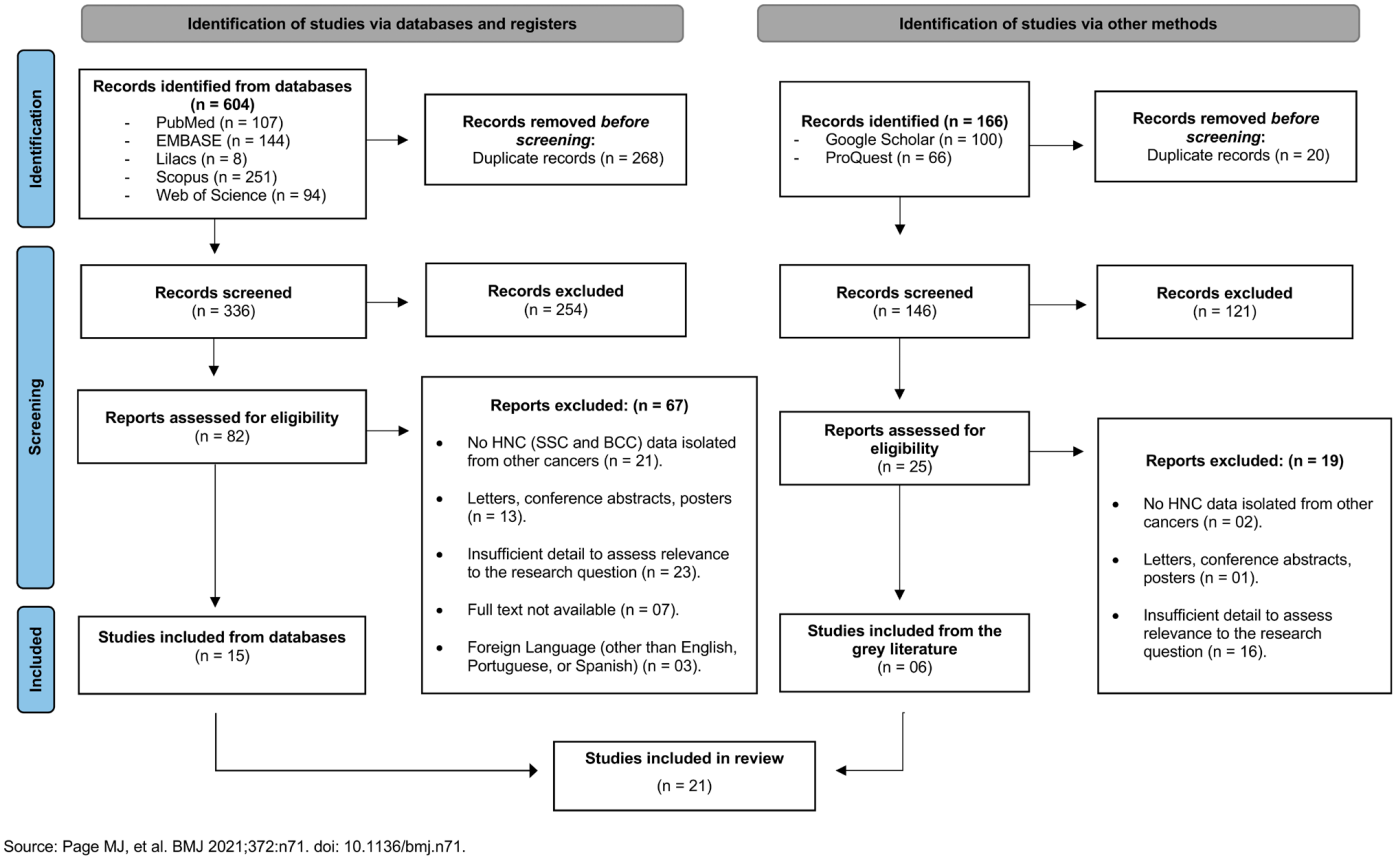
PRISMA 2020 flow diagram for new systematic reviews which included searches of databases, registers and other sources.

### 3.2 Characteristics of Sources of Evidence

The included studies were published between 2006 and 2024 (Li et al. 2022; Chang et al. 2022; Musa et al. 2024; Goswami and Gupta 2019; Becker et al. 2006; Reis et al. 2021, 2020; Menezes 2023; Ludin et al. 2018; Philipp et al. 2022; Miller 2020; Caldin et al. 2021; Khokhar et al. 2022; Wallace 2013, 2019; Bingo et al. 2020; Popescu and Păun 2017; Agarwal et al. 2010; Santana et al. 2008; Zhu et al. 2024; Wong et al. 2006). The highest number of publications occurred in 2022 (19%, *n* = 4) (Li et al. 2022; Chang et al. 2022; Philipp et al. 2022; Khokhar et al. 2022) followed by 2021 (9.5%, *n* = 2) (Reis et al. 2021; Caldin et al. 2021), 2019 (9.5%, *n* = 2) (Goswami and Gupta 2019; Wallace 2013), and 2020 (9.5%, *n* = 2) (Reis et al. 2020; Bingo et al. 2020). Brazil contributed the largest proportion of studies (23.8%) (Reis et al. 2021, 2020; Menezes 2023; Caldin et al. 2021; Santana et al. 2008), followed by the United States (19%) (Miller 2020; Wallace 2013, 2019; Agarwal et al. 2010), China (19%) (Li et al. 2022; Chang et al. 2022; Zhu et al. 2024; Wong et al. 2006), and Malaysia (9.5%) (Musa et al. 2024; Ludin et al. 2018). Cross‐sectional (Agarwal et al. 2010; Chang et al. 2022; Reis et al. 2021, 2020; Santana et al. 2008; Zhu et al. 2024) and qualitative (Caldin et al. 2021; Khokhar et al. 2022; Goswami and Gupta 2019; Wallace 2013, 2019; Bingo et al. 2020) studies were the most frequent designs (28.6% each, (*n* = 6). Cohort studies accounted for 14.3% ((*n* = 3) (Becker et al. 2006; Musa et al. 2024; Ludin et al. 2018). Clinical trials (Li et al. 2022), literature reviews (Popescu and Păun 2017), and case‐control studies (Menezes 2023) were less common, each representing 4.8% (*n* = 1). Excluding the review, all studies were primary research involving patient assessments (*n* = 4008 participants). The mean participant age was 56.4 years, with sample sizes from 15 to 1234. Most studies (90.5%) included both sexes; one study included only males (Agarwal et al. 2010), and another only female (Khokhar et al. 2022).

Eighteen studies reported anatomical sites (Li et al. 2022; Chang et al. 2022; Musa et al. 2024; Goswami and Gupta 2019; Reis et al. 2021, 2020; Menezes 2023; Ludin et al. 2018; Philipp et al. 2022; Miller 2020; Caldin et al. 2021; Khokhar et al. 2022; Wallace 2013, 2019; Bingo et al. 2020; Agarwal et al. 2010; Zhu et al. 2024; Wong et al. 2006), six of which focused exclusively on the oral cavity (Musa et al. 2024; Goswami and Gupta 2019; Philipp et al. 2022; Caldin et al. 2021; Khokhar et al. 2022; Wong et al. 2006). Eleven studies classified patients by tumor Stage (I–IV) (Chang et al. 2022; Musa et al. 2024; Goswami and Gupta 2019; Becker et al. 2006; Reis et al. 2021, 2020; Menezes 2023; Philipp et al. 2022; Miller 2020; Santana et al. 2008; Wong et al. 2006), one by stages II–IV (Wallace 2013), and one by the presence of metastasis (Li et al. 2022). Eight studies did not report staging (Ludin et al. 2018; Caldin et al. 2021; Khokhar et al. 2022; Wallace 2019; Bingo et al. 2020; Popescu and Păun 2017; Agarwal et al. 2010; Zhu et al. 2024). Treatment reporting was inconsistent: 23.8% did not specify treatment modalities (Khokhar et al. 2022; Wallace 2013, 2019; Popescu and Păun 2017; Santana et al. 2008). Surgery alone or in combination with radiotherapy/chemotherapy (Musa et al. 2024; Ludin et al. 2018; Wong et al. 2006) was reported in 14.3% of studies each. Radiotherapy, chemotherapy, and surgery (in unspecified sequence) (Reis et al. 2021, 2020; Agarwal et al. 2010) accounted for 14.3%, while radiotherapy alone was reported in 9.5% (Becker et al. 2006; Zhu et al. 2024).

Across studies, spirituality and religiosity consistently emerged as important coping resources for individuals with HNC (Table 1). Li et al. (2022) demonstrated that a holistic intervention incorporating spiritual care (interventions) significantly improved patient outcomes, reducing psychological distress and unmet needs. Patients with lower levels of spiritual well‐being reported greater discomfort and embarrassment regarding postsurgical appearance changes (Reis et al. 2021). Religious faith was frequently used by socioeconomically disadvantaged patients, offering hope and emotional relief, though younger patients tended to rely less on spirituality and more on biomedical approaches. The absence of religious belief was associated with despair and reduced coping capacity (Popescu and Păun 2017). Some studies examined the association between religiosity and mortality risk among patients with HNC (Menezes 2023; Khokhar et al. 2022; Wong et al. 2006).

**TABLE 1:** Studies included in the scoping review.

Author	Study design	Objective	Conclusion
Becker et al. (2006)	Cohort study using data from a previous RCT (MF‐4449); QoL assessed at four time points. Religious belief assessed by: “Do you have a belief that gives you comfort?”	To assess the role of religious belief in coping with radiotherapy in HNC patients.	Religious faith was associated with better coping, less pain, and reduced fear.
Musa et al. (2024)	Secondary analysis of health‐related quality of life data from 317 oral cancer patients using FACT‐H&N; multiple linear regression at baseline, 1‐ and 3‐months posttreatment	To examine the role of social support and spiritual aspects in predicting psychosocial well‐being among Malaysian oral cancer patients with restricted oral function.	Spiritual aspects interact with other predictors; integrating emotional support from family, caregivers, and friends alongside spiritual care is essential to optimize outcomes and improve quality of life during survivorship.
Caldin et al. (2021)	Cohort study of 81 newly diagnosed head and neck cancer patients; QoL assessed pre‐treatment and 6 months posttreatment using Cancer Survivor and QLQ‐H&N35; descriptive and inferential statistics.	To determine and compare pre‐ and posttreatment quality of life in patients with head and neck cancer.	Among all QoL dimensions, spiritual well‐being—especially “hopefulness”—showed the highest mean scores both before and after treatment.
Li et al. (2022)	Randomized controlled trial with follow‐ups up to 6 months post‐discharge.	To test a holistic care model (TIR) for radiotherapy patients.	The TIR‐based care program significantly improved patient information needs, psychological and physical symptoms, quality of healthcare services and support, including religious and spiritual care, while reducing psychological distress and pain compared to the control group.
Ludin et al. (2018)	Cross‐sectional study of African American HNC survivors; coping strategies assessed by the Ways of Helping Questionnaire.	To describe coping strategies used by older African American HNC survivors and examine associations with quality of life and social closeness.	Survivors who relied on God and/or family and friends reported better quality of life. Those using self‐reliance or depending on others for physical care had lower quality of life and social functioning.
Chang et al. (2022)	Cross‐sectional study comparing high‐ and low‐demoralization groups regarding sociodemographic characteristics, QoL, and spiritual needs.	To provide insights into the potential links between demoralization in oral cancer patients and its effects on spiritual needs, quality of life, and suicidal ideation.	High demoralization was associated with lower satisfaction of spiritual needs and negatively correlated with quality of life and spiritual well‐being. Positive attitudes, love, seeking life's meaning, and peace of mind were linked to lower demoralization.
Reis et al. (2021)	Cross‐sectional study of 202 patients; psychosocial, demographic, and clinical data collected; QoL assessed by FACT‐HN and UW‐QOL; religiosity by DUREL; spirituality by FACIT‐Sp12.	To investigate associations between religiosity, spirituality, and quality of life in patients with visible sequelae following head and neck cancer surgery.	Higher quality of life scores was observed in patients with greater religiosity, males, those living with family, patients without cervical sequelae, and those who did not receive chemotherapy or radiotherapy.
Reis et al. (2020)	Cross‐sectional study collecting sociodemographic, psychosocial, clinical, and cancer‐related behavior data; appearance concerns assessed by Derriford Appearance Scale (DAS24) in surgically treated HNC patients with visible sequelae.	To investigate the association of religiosity and spiritual well‐being with appearance concerns in patients with visible sequelae from head and neck cancer surgery.	Higher spiritual well‐being was independently associated with lower appearance concerns, indicating a positive role in coping with visible sequelae. These challenges affect patients, families, social networks, and healthcare systems.
Philipp et al. (2022)	Cross‐sectional study assessing coping strategies, social networks, and social support among 22 head and neck cancer patients using questionnaires and clinical records.	To assess coping strategies, social networks, and social support among head and neck cancer patients.	Coping is strongly influenced by problem‐focused strategies and religiosity, which complement each other. Insufficient social support reveals gaps in patients' networks, indicating the need to enhance social resources.
Miller (2020)	Multicenter cross‐sectional study using Distress Thermometer (DT) and Hospital Anxiety and Depression Scale (HADS).	To estimate the prevalence of clinically significant baseline distress in Chinese patients with head and neck squamous cell carcinoma prior to initial radiotherapy, and to identify predictors of distress.	Religious belief was significantly associated with lower emotional distress. Patients with religious beliefs reported less clinically significant distress, suggesting a protective role of religiosity.
Popescu and Păun (2017)	Qualitative study using semi‐structured interviews addressing sociodemographic data and patient perceptions including: “What changed in your life with the diagnosis of head and neck cancer?” and “How do you perceive your current health status?”	To analyze adaptation modes related to self‐concept and role function in patients undergoing treatment for oral cavity cancer.	Patients exhibited both negative and positive adaptations: many reported low self‐esteem and sadness due to physical changes, while others experienced improved self‐perception supported by religious beliefs and hope.
Agarwal et al. (2010)	Qualitative study using open‐ended interviews and narrative analysis with a storytelling approach.	To explore how Islamic beliefs, spiritual practices, and fatalism may act as barriers to oral cancer diagnosis in Pakistani women and understand their disease experience within the sociocultural context.	Religious fatalism shaped patients' understanding of oral cancer, with belief that Allah both causes and cures the disease, contributing to delayed care‐seeking. Integrating traditional healers and improving communication may reduce delays and improve outcomes.
Goswami and Gupta (2019)	Qualitative study using in‐depth interviews with posttreatment oral cancer patients, exploring challenges and coping strategies.	To achieve a deeper understanding of patients' concerns regarding the diagnosis, treatment, and coping processes of oral cancer.	Family support is essential for coping, and spiritual well‐being significantly contributes to quality of life, closely related to psychological and physical distress in advanced cancer patients.
Zhu et al. (2024)	Qualitative study using in‐depth interviews at diagnosis and after treatment completion, exploring patient worries and fears.	To explore the treatment decision‐making experiences of adults newly diagnosed with head and neck squamous cell carcinoma, including laryngeal, esophageal, and oral cancers.	Spirituality, grounded in hope and self‐reflection, helped patients preserve inner identity and transcend illness. Spiritual practices such as prayer, meditation, and service were key coping strategies fostering resilience. Participation in 12‐step programs further supported spiritual growth and helped patients manage fears and treatment decisions.
Wong et al. (2006)	Qualitative study using unstructured interviews with South African head and neck cancer patients.	To describe the lived experiences of South African patients with head and neck cancer.	Participants experienced significant life changes due to pain, eating and communication difficulties, and social stigma. Coping was supported by family, friends, and faith. Hope, prayer, and positivity were key coping resources.
Santana et al. (2008)	Qualitative study with structured interviews during treatment, including health, time perspective, coping, and decision‐making measures.	To explore the treatment experience of older adults newly diagnosed with head and neck cancer, examining time and age influences on coping, and to develop a model and strategies to improve treatment experience.	Patients undergoing oral cavity cancer treatment experience a complex emotional and spiritual journey. Initial responses included seeking medical help and spiritual practices, with faith as a key coping mechanism. Despite early anger, many reported emotional and spiritual growth marked by increased spiritual awareness, forgiveness, acceptance, and mortality understanding. Recovery involved redefining self through hope, reflection, and spiritual resilience, helping patients transcend illness.
Khokhar et al. (2022)	Retrospective study recording patient characteristics, oncological variables, posttreatment impairments, QoL, and psychosocial factors.	To evaluate health‐related quality of life (HRQoL) and psychosocial variable outcomes in patients treated for oral squamous cell carcinoma.	Women showed greater reliance on religiosity and spiritual coping, which was linked to higher disease acceptance despite more depression and anxiety. Spiritual coping buffered distress and supported QoL. Spirituality and religiosity play key roles in psychosocial resilience and quality of life.
Jetan et al. (2023)	Retrospective study reviewing electronic health records of 262 head and neck cancer patients assessing factors related to treatment nonadherence.	To examine whether demographic, clinical, physical, and psychological symptoms are associated with nonadherence to radiotherapy among HNC patients.	Patients with higher spiritual well‐being were less likely to be nonadherent. Spiritual well‐being may protect against nonadherence.
Wallace (2019)	Retrospective review of 1010 oral cancer patient charts diagnosed from 1995 to 2002, with follow‐up until end of 2004.	To determine survival rates and assess socio‐demographic factors influencing survival in oral cancer patients.	Patients without religious belief had a poorer prognosis, with a relative risk of 2.057, and religious belief remained a significant prognostic factor after adjusting for clinical variables.
Bingo et al. (2020)	Case–control longitudinal study comparing weekly acupuncture intervention during radiotherapy versus no intervention.	To evaluate the effect of acupuncture, spirituality, religious coping, personal beliefs, and quality of life on survival in head and neck cancer patients.	Increased survival was associated with greater organizational religiosity (e.g., religious affiliation and frequent temple attendance). Thus, enhanced survival in these patients was linked to superior physical and psychological quality of life as well as higher levels of organizational religiosity.
Menezes (2023)	Literature review on coping mechanisms in head and neck cancer patients.	To explore how patients with head and neck cancer psychologically and socially cope with the disease, focusing on the role of religious and spiritual support.	Spirituality is a key coping mechanism providing hope. Lack of religious faith may increase suffering and impede coping, sometimes causing neglect of medical treatment. For advanced disease, integrating spiritual beliefs with medical and social support is crucial, especially with limited family support. Physicians should respect cultural, social, and religious contexts, particularly with low health literacy or socioeconomic challenges.

The FACIT‐Sp‐12 and FACT‐H&N (Functional Assessment of Cancer Therapy—Head and Neck) were the most frequently used instruments, each appearing in three articles (Musa et al. 2024; Reis et al. 2021, 2020). The DUREL (Duke University Religiosity Index) was the most commonly used measure of religiosity (Reis et al. 2021, 2020; Menezes 2023). The Edmonton Symptom Assessment System–Revised–Constipation and Spiritual Symptoms (ESAS‐r‐CSS) was another instrument used to assess spiritual dimensions of symptom burden (Miller 2020). In addition, studies employed various psychological, QoL, and coping measures (see Table S2).

### 3.3 Synthesis of Results

#### 3.3.1 Spirituality

Spirituality consistently emerged as a key coping mechanism among individuals with HNC. The pursuit of meaning, positive attitudes, inner peace, love, and religious beliefs were identified as essential contributors to psychological resilience and emotional well‐being (Chang et al. 2022; Musa et al. 2024; Ludin et al. 2018; Reis et al. 2020; Philipp et al. 2022; Agarwal et al. 2010). Spiritual practices such as prayer, meditation, and self‐reflection were reported to help patients sustain hope and find meaning during treatment (Wallace 2019). Across the included studies, spirituality was associated with improved QoL in 28.6% of studies (*n* = 6) (Chang et al. 2022; Musa et al. 2024; Goswami and Gupta 2019; Ludin et al. 2018; Reis et al. 2020; Agarwal et al. 2010) and with enhanced coping and emotional resilience in 14.3% (*n* = 3) (Li et al. 2022; Wallace 2019; Zhu et al. 2024). Detailed outcomes are provided in Table S2.

Coping strategies frequently involved reliance on faith in God, social support, and altruistic behaviors toward others. Greater reliance on divine help was linked to better physical function and overall QoL (Agarwal et al. 2010). Emotional and informational support correlated strongly (*r* = 0.785, *p* < 0.01), as did problem‐focused coping and religiosity (*r* = 0.579, *p* < 0.05) (Santana et al. 2008). Women demonstrated greater acceptance of oral cancer when using religious coping (Philipp et al. 2022). Patients with visible deformities, who reported higher religiosity, male sex, family support, and absence of cervical sequelae, reported better QoL (Reis et al. 2020).

### 3.4 Treatment Time Assessment

Most studies assessed spirituality in the posttreatment phase (38.1%, *n* = 8) (Goswami and Gupta 2019; Reis et al. 2021, 2020; Menezes 2023; Philipp et al. 2022; Bingo et al. 2020; Agarwal et al. 2010; Wong et al. 2006), while six (28.6%) evaluated both pre‐ and post‐treatment (Li et al. 2022; Musa et al. 2024; Ludin et al. 2018; Caldin et al. 2021; Wallace 2013, 2019). Four studies (19.0%) were conducted during treatment (Chang et al. 2022; Miller 2020; Khokhar et al. 2022), two (9.5%) focused on pretreatment (Santana et al. 2008; Zhu et al. 2024), and one (4.8%) addressed palliative care (Popescu and Păun 2017). Main outcomes by timepoint are summarized in Table 2.

**TABLE 2:** Outcomes related to spirituality according to timepoint.

Time point	Studies (*n*)	Studies (%)	Outcome (*n*)
*Influence of spirituality and religiosity on coping with illness*			
Before treatment	1.00	4.8	Coping is influenced by religiosity (1)
During treatment	2.00	9.5	Religious belief improves coping (1); Coping is influenced by religiosity (1)
Posttreatment	2.00	9.5	Religious belief improves coping (1); Coping is influenced by religiosity (1)
Before and post‐ treatment	1.00	4.8	Religious belief improves coping (1)
Palliative	1.00	4.8	Coping is influenced by religiosity (1)
*Association of spirituality with clinical outcomes and prognosis*			
Posttreatment	2.00	9.5	Religious belief impacts on better prognosis (2)
During treatment	1.00	4.8	Religious belief improves coping and treatment adherence (1)
*Spirituality impacts on emotional well‐being, quality‐of‐life, and psychological distress*			
Before treatment	1.00	4.8	Religious or spiritual support reduces distress in coping with disease (1)
Before and post‐ treatment	5.00	23.8	Religious or spiritual support reduces distress in coping with disease (1); Spirituality improves coping and emotional resilience (1); Spiritual aspects improve quality‐of‐life (QoL) (3)
Posttreatment	5.00	23.8	Spiritual aspects contribute to improved quality‐of‐life (QoL) (5)

Before treatment, religiosity and social support acted as protective coping strategies, helping patients manage fear and emotional distress (Santana et al. 2008; Zhu et al. 2024). During treatment, spirituality was associated with treatment adherence, informed decision‐making, and alleviation of psychological burden. Respecting cultural beliefs, including the involvement of traditional healers, was linked with better engagement and outcomes. Hope, meaning, and positive attitudes reduced demoralization in oral cancer patients (Chang et al. 2022; Miller 2020; Caldin et al. 2021; Khokhar et al. 2022). After treatment, prayer, spiritual support, and social connectedness were central coping strategies, contributing to improved QoL (Goswami and Gupta 2019; Agarwal et al. 2010). In palliative care, spirituality provided essential comfort and hope, particularly for patients with limited family support, while lack of faith was associated with greater suffering (Popescu and Păun 2017).

Studies comparing pre‐ and posttreatment periods showed that overall QoL declined from moderate pretreatment levels (6.22 ± 1.80) to lower levels following treatment (4.84 ± 1.16). Psychological well‐being had the lowest mean score (5.54 ± 1.83), while spiritual well‐being had the highest (7.03 ± 1.88), followed by physical (6.75 ± 1.66) and social domains (6.54 ± 1.89)14. Low QoL and unmet spiritual needs (< 3.7) predicted higher demoralization in hospitalized oral cancer patients (Chang et al. 2022). Greater spiritual well‐being was associated with fewer appearance‐related concerns, particularly among men and those with less severe disfigurement or not receiving radiotherapy or chemotherapy (Reis et al. 2021). Consistent findings were observed with FACIT‐Sp‐12, which showed a moderate to strong correlation with spiritual well‐being (*r* = 0.59; *p* < 0.001) (Reis et al. 2020).

Across stages, patients used prayer and reflection to manage distress, reported spiritual and emotional growth during treatment, and described renewed purpose after recovery (Khokhar et al. 2022; Wallace 2013). Activities such as volunteering and structured programs supported this process (Wallace 2019). Higher religiosity was also associated with lower distress before radiotherapy (Distress Thermometer OR = 0.291; HADS OR = 0.316) (Zhu et al. 2024).

### 3.5 Spiritual Care

Support intervention groups, including 12‐step programs, helped patients make treatment decisions, reflect on their experiences, and develop personal acceptance (Wallace 2019). Integrating spirituality into care was linked to fewer highly invasive procedures in palliative settings, and clinicians were encouraged to respect patients' social, cultural, and religious beliefs, especially among those with low health literacy or socioeconomic disadvantage (Popescu and Păun 2017). Collaboration with traditional healers also helped reduce fatalistic beliefs, promote earlier diagnosis, and improve oral cancer management (Khokhar et al. 2022). Commonly recommended strategies included cognitive‐behavioral approaches, education and support groups, interventions for body‐image concerns, encouragement of emotional expression, and support for family members and caregivers (Santana et al. 2008).

The TIR *(Timing ir right)* model was described as a comprehensive framework addressing physical, emotional, informational, and spiritual needs across the cancer pathway, helping reduce distress and improve QoL (Li et al. 2022). Before radiotherapy, key interventions involved emotional support, clear communication, anxiety reduction, and strengthening social support. During radiotherapy, patients benefited from expressing concerns, accessing educational materials, receiving individualized follow‐up, and daily well‐being checks. After discharge, structured telephone follow‐ups supported ongoing adjustment and treatment adherence (Li et al. 2022).

## 4 Discussion

Spirituality represents an important dimension of the illness experience for patients with HNC and has been associated with emotional responses, coping behaviors, treatment engagement, and overall QoL across the cancer care continuum. Although spirituality is widely recognized as a resource for patients facing life‐threatening illness, our findings show that its influence varies by treatment stage and closely interacts with psychological well‐being, social support, cultural context, and care delivery.

Spirituality is more closely associated with inner peace and life purpose than with religious affiliation or ritual observance, although these constructs often overlap in oncology patients (Whitford and Olver 2012; Jetan et al. 2023). Previous evidence indicates that religiosity serves as a coping resource and is associated with greater well‐being (Nagy et al. 2024; Mollica et al. 2016). Religiosity has been described as a coping resource (Agarwal et al. 2010; Santana et al. 2008) and has been associated with reduced psychological distress (Zhu et al. 2024) and, in some studies, has also been linked to survival outcomes (Menezes 2023; Wong et al. 2006), and better management of HNC treatment‐related side effects (Becker et al. 2006). Findings from this review emphasize spirituality and religiosity as important resources for patients with HNC. Spiritual care not only addresses emotional and existential suffering but also respects cultural and religious values that influence patients' experiences and decision‐making (Balboni et al. 2022).

Spirituality also emerged as a relevant dimension of patient well‐being before, during, and after cancer therapy. Before initiating treatment, religiosity and social support were reported as protective factors that were associated with lower levels of fear, emotional distress, and demoralization (Santana et al. 2008; Zhu et al. 2024). At diagnosis, prayer and reflection often appeared as immediate coping responses that helped patients manage uncertainty and anticipate treatment demands (Caldin et al. 2021; Khokhar et al. 2022; Wallace 2013). During treatment, spirituality was positively associated with treatment adherence, collaborative decision making, and psychological resilience while treatment‐related toxicities produced lower distress in patients reporting greater spirituality (Chang et al. 2022; Miller 2020; Caldin et al. 2021; Khokhar et al. 2022). Respecting cultural and religious beliefs, including working with traditional healers, helped counter fatalism and encouraged early cancer management (Khokhar et al. 2022). After treatment, spiritual growth became more prominent, with reports of renewed purpose, strengthened identity, acceptance, compassion, and broader social connectedness (Goswami and Gupta 2019; Wallace 2013; Agarwal et al. 2010). In palliative settings, spirituality appeared to support comfort, meaning, and adaptive coping, particularly when reinforced by psychosocial and family resources (Popescu and Păun 2017; Santana et al. 2008). Taken together, these findings suggest that spirituality and social support may function as dynamic resources whose relevance changes over time, reinforcing the importance of integrating spiritual care across the different stages of the HNC trajectory.

Across studies, spiritual well‐being was consistently associated with better psychosocial outcomes. Higher scores correlated with fewer appearance‐related concerns (Reis et al. 2021) and greater overall well‐being, including moderate to strong associations with FACIT‐Sp‐12 (Reis et al. 2020). Importantly, pre‐post comparisons showed a decline in overall QoL, from moderate pre‐treatment levels to lower levels after treatment, while spiritual well‐being remained the highest domain (Ludin et al. 2018). Low QoL and unmet spiritual needs predicted high demoralization, highlighting the clinical relevance of addressing existential concerns (Chang et al. 2022).

Several studies in this review reported associations between religiosity and mortality risk in HNC (Menezes 2023; Khokhar et al. 2022; Wong et al. 2006). These findings suggest that religiosity may be associated with survival not only through processes of spiritual meaning‐making but also through indirect pathways such as treatment adherence, health‐related behaviors, social support networks, and psychological resilience (Menezes 2023; Khokhar et al. 2022; Wong et al. 2006). However, the interpretation and impact of religiosity on health outcomes may vary according to sociocultural context.

This review included studies conducted in diverse sociocultural contexts across the world and included studies from Brazil (Reis et al. 2021, 2020; Menezes 2023; Caldin et al. 2021; Santana et al. 2008), China (Li et al. 2022; Chang et al. 2022; Zhu et al. 2024; Wong et al. 2006), the United States (Miller 2020; Wallace 2013, 2019; Agarwal et al. 2010), Malaysia (Musa et al. 2024; Ludin et al. 2018), Germany (Becker et al. 2006), Romania (Popescu and Păun 2017), India (Goswami and Gupta 2019), South Africa (Bingo et al. 2020), Pakistan (Khokhar et al. 2022), and the German–Austrian–Swiss region of central Europe (Philipp et al. 2022). This geographic diversity reflects heterogeneous patient populations, social structures, religious traditions, and healthcare systems, all of which may impact the ways in which spirituality and religiosity are experienced and expressed by patients with HNC. In some sociocultural contexts, fatalistic religious beliefs may influence illness interpretation and patterns of healthcare engagement. For example, Khokhar et al. (2022) reported that Islamic fatalism shaped patients' understanding of oral cancer, with some individuals believing that Allah both causes and cures the disease, a perception that could delay care‐seeking behaviors. In addition, a study conducted in the German–Austrian–Swiss context was the only one to examine gender differences, showing that women reported greater reliance on religious coping and higher acceptance of the disease despite greater psychological distress. In the same population, men experienced higher levels of treatment‐related functional impairment and lower levels of social integration (Philipp et al. 2022). Similarly, findings from a Chinese study (Li et al. 2025) suggest that coping self‐efficacy may also vary according to gender and social context, highlighting ways in which broader psychosocial and cultural factors may shape patient coping mechanisms, including spiritual and meaning‐based support (Mollica et al. 2016). These findings suggest that spirituality in patients with HNC is not a uniform construct, but a culturally mediated experience shaped by religious traditions, gender‐related considerations, societal norms, family structures, social support networks, and the interaction between biomedical care and belief systems.

Clinical guidelines strongly recommend providing spiritual care (Miller et al. 2025), yet its implementation was rarely addressed in the included studies. Many oncology professionals lack adequate training, confidence, and institutional support to provide spiritual care, facing barriers such as limited knowledge, uncertainty about initiating discussions, religious differences, and time constraints (Miller et al. 2025; Ichihara et al. 2024). Wenham et al. (2021) recommends strengthening professionals' self‐reflection, respecting patients' beliefs, and routinely screening for spiritual needs with referral to specialists when appropriate, aligning with Delphi‐based guidance on spiritual care (Balboni et al. 2022). Suggested practices include asking whether spirituality or faith influences coping or illness understanding and ensuring someone is available for related discussions (Balboni et al. 2022). A clinical trial also introduced a nursing training program based on the SpiPas‐SCP‐N model, which assesses spiritual pain across relationship, autonomy, and temporality domains and guides interventions to promote meaning, peace, dignity, acceptance, symptom relief, and coordinated team support (Wise et al. 2018).

The TIR model provided a comprehensive framework for integrating physical, emotional, informational, and spiritual support throughout treatment (Li et al. 2022). Pre‐radiotherapy interventions emphasized emotional support and anxiety reduction, while radiotherapy‐focused strategies included guided expression of concerns, educational materials, and individualized follow‐up. Post‐discharge, structured telephone monitoring helped sustain adaptation and adherence (Li et al. 2022). Collectively, these interventions suggest that addressing spiritual needs in oncology care is feasible, culturally adaptable, and associated with improved QoL and reduced psychological distress (Miller et al. 2025). Evidence from a meta‐analysis of psychosocial interventions also indicates that such approaches can significantly improve spiritual well‐being among cancer patients (McLouth et al. 2021).

While this review is comprehensive, it includes inherent limitations related to the component studies that must be considered when interpreting the findings. Two studies meeting inclusion criteria included study populations in which patients with skin cancer (*n* = 2) and esophageal cancer (*n* = 2) were analyzed with HNC populations. In these studies, results were not reported by tumor type, making it impossible to extract data exclusive to HNC patients. These studies were nevertheless retained due to similarities in clinical burden and treatment‐related functional impairments between patient groups. Additionally, conceptualization and measurement of spirituality varied across studies, reflecting differences in the theoretical frameworks and assessment instruments used to characterize it. Finally, the sociocultural diversity of the study settings, together with the inconsistent reporting of clinical variables (such as tumor stage and treatment protocols), should be considered when interpreting comparability and generalizability of study findings.

## 5 Conclusion

Spirituality and religiosity were frequently associated with coping, emotional well‐being, and QoL in patients with HNC. Their expression and impact appear to be shaped by sociocultural factors, including religious traditions, fatalistic beliefs, family and social structures, community support networks, and the coexistence of biomedical and traditional healing practices. Because spiritual needs and coping resources vary across the treatment continuum from diagnosis to survivorship and end‐of‐life care, spiritual care should be tailored to each of the patient journey trajectory.

## Author Contributions

L.W.B. and C.M.P. contributed to methodology, data collection, formal analysis, investigation, visualization, and drafting of the original manuscript. A.R.S.‐S. and H.M.D. were responsible for conceptualization, project administration, and supervision. M.D.M., A.C.P.‐R., D.D, T.P.S., and H.M.C. contributed to validation and critically revised and edited the final version of the manuscript.

## Funding

The authors acknowledge financial support from the São Paulo Research Foundation (FAPESP), grant 2024/20694‐1, and the Brazilian National Council for Scientific and Technological Development (CNPq), grant 201672/2025‐1.

## Conflicts of Interest

The authors declare no conflicts of interest.

## Supporting information


**Table S1:** Preliminary search strategy.

**Table S2:** Measurement instruments.

## Data Availability

Data available on request from the authors.

## References

[odi70335-bib-0001] Agarwal, M. , J. B. Hamilton , J. L. Crandell , and C. E. Moore . 2010. “Coping Strategies of African American Head and Neck Cancer Survivors.” Journal of Psychosocial Oncology 28, no. 5: 526–538. 10.1080/07347332.2010.498456. 20730663

[odi70335-bib-0002] Balboni, T. A. , T. J. Vanderweele , S. D. Doan‐Soares , et al. 2022. “Spirituality in Serious Illness and Health.” Journal of the American Medical Association 328, no. 2: 184–197. 10.1001/jama.2022.11086. 35819420

[odi70335-bib-0003] Becker, G. , F. Momm , C. Xander , et al. 2006. “Religious Belief as a Coping Strategy: An Explorative Trial in Patients Irradiated for Head‐And‐Neck Cancer.” Strahlentherapie und Onkologie 182, no. 5: 270–276. 10.1007/s00066-006-1533-9. 16673060

[odi70335-bib-0004] Bingo, S. A. M. , J. E. Maree , and J. J. M. Jansen van Rensburg . 2020. “Living With Cancer of the Head and Neck: A Qualitative Inquiry Into the Experiences of South African Patients.” European Journal of Cancer Care 29, no. 2: e13205. 10.1111/ecc.13205. 31829489

[odi70335-bib-0005] Caldin, L. N. , L. A. C. Medina , R. A. Silva , et al. 2021. “Self‐Concept and Role Function in Patients With Head and Neck Cancer.” Acta Paulista de Enfermagem 34, no. 2: eAO00892. 10.37689/acta-ape/2021AO00892.

[odi70335-bib-0006] Chang, T. G. , P. C. Huang , C. Y. Hsu , and T. T. Yen . 2022. “Demoralization in Oral Cancer Inpatients and Its Association With Spiritual Needs, Quality of Life, and Suicidal Ideation: A Cross‐Sectional Study.” Health and Quality of Life Outcomes 20, no. 1: 19. 10.1186/s12955-022-01962-6. PMC8976948 35366908

[odi70335-bib-0007] Ferri, A. , G. Lilloni , G. Molteni , et al. 2024. “The Psychosocial Needs of Head and Neck Cancer Patients: A Multicenter Study.” European Archives of Oto‐Rhino‐Laryngology 281, no. 9: 4913–4920. 10.1007/s00405-024-08680-3. 38704510

[odi70335-bib-0008] Goswami, S. , and S. Gupta . 2019. “How Patients of Oral Cancer Cope With the Impact of the Disease: A Qualitative Study in Central India.” Indian Journal of Palliative Care 25, no. 1: 103–109. 10.4103/IJPC.IJPC_118_18. PMC6388589 30820111

[odi70335-bib-0009] Hu, Q. , S. Lv , X. Wang , P. Pan , W. Gong , and J. Mei . 2025. “Global Burden and Future Trends of Head and Neck Cancer: A Deep Learning‐Based Analysis (1980–2030).” PLoS One 20, no. 4: e0320184. 10.1371/journal.pone.0320184. PMC11981659 40203229

[odi70335-bib-0010] Ichihara, K. , C. Nishiyama , K. Kiyohara , et al. 2024. “Nursing Care for Spiritual Pain in Terminal Cancer Patients.” Journal of Pain and Symptom Management 67, no. 2: 126–137. 10.1016/j.jpainsymman.2023.10.016 37852454

[odi70335-bib-0011] Jetan, M. , A. Daifallah , M. K. Rabayaa , et al. 2023. “The Impact of Spiritual Well‐Being on the Quality of Life of Cancer Patients: A Cross‐Sectional Study.” Integrative Cancer Therapies 22: 15347354231210841. 10.1177/15347354231210841 PMC10647945 37961876

[odi70335-bib-0012] Khokhar, M. A. , B. Gibson , M. Winslow , and J. Owens . 2022. “Oral Cancer Is a Punishment for My Sins: Oral Histories of Oral Cancer, Fatalism and Islamic Religious Beliefs in Pakistan.” Journal of Religion and Health 61, no. 6: 4337–4351. 10.1007/s10943-022-01585-7. 35639222

[odi70335-bib-0013] Li, S. , Z. Gao , Y. Xu , X. Zhong , and X. Bai . 2025. “Self‐Efficacy for Coping With Cancer and Quality of Life in Head and Neck Cancer Patients: A Cross‐Sectional Study.” Scientific Reports 15: 7056. 10.1038/s41598-025-91360-x. PMC11868415 40016515

[odi70335-bib-0014] Li, T. , Y. P. Zhang , J. J. Kong , M. Kong , G. Meng , and W. X. Shi . 2022. “Effect of ‘Timing It Right’ on Comprehensive Unmet Needs and Psychological Pain in Patients With Head and Neck Cancer Undergoing Radiotherapy: A Randomized Controlled Trial.” Supportive Care in Cancer 30, no. 8: 6573–6582. 10.1007/s00520-022-07072-9. 35488009

[odi70335-bib-0015] Ludin, S. M. , C. A. C. Abdullah , and K. Abdullah . 2018. “Quality of Life in Pre‐ and Post‐Treatment Among Head and Neck Cancer Survivors at a Tertiary Centre in Malaysia.” Makara Journal of Health Research 22, no. 2: 67–72. 10.7454/msk.v22i2.9267.

[odi70335-bib-0016] McLouth, L. E. , C. G. Ford , J. E. Pustejovsky , et al. 2021. “A Systematic Review and Meta‐Analysis of Effects of Psychosocial Interventions on Spiritual Well‐Being in Adults With Cancer.” Psycho‐Oncology 30, no. 2: 147–158. 10.1002/pon.5562. PMC8485897 34602807

[odi70335-bib-0017] Menezes, A. S. 2023. “Acupuntura, Religiosidade, Qualidade de Vida e Sobrevida de Pacientes com Carcinoma de Células Escamosas de Cabeça e Pescoço [Dissertation].” Montes Claros (Brazil): Universidade Estadual de Montes Claros.

[odi70335-bib-0018] Miller, J. L. 2020. “Predictors of Nonadherence to Radiation Therapy Schedules Among Head and Neck Cancer Patients [Dissertation].” Chicago (IL): The Chicago School of Professional Psychology.

[odi70335-bib-0019] Miller, M. , M. Meyers , K. Krainak , and S. P. Lewis . 2025. “Interventions to Support Spirituality Among Adults With Cancer.” Supportive Care in Cancer 33: 742. 10.1007/s00520-025-09787-x. PMC12317903 40751754

[odi70335-bib-0020] Mollica, M. A. , W. Underwood , G. G. Homish , et al. 2016. “Spirituality Is Associated With Better Prostate Cancer Treatment Decision Making Experiences.” Journal of Behavioral Medicine 39, no. 1: 161–169. 10.1007/s10865-015-9662-1. 26243642

[odi70335-bib-0021] Musa, M. F. C. , J. G. Doss , W. M. N. Ghani , S. N. Rogers , S. M. Ismail , and R. A. Rahman . 2024. “The Roles of Social Support and Spiritual Aspect in Predicting the Association Between Oral Functioning and Psychosocial Well‐Being Among Oral Cancer Patients.” Malaysian Journal of Medicine and Health Sciences 20, no. 4: 63–71. 10.47836/mjmhs.20.4.9.

[odi70335-bib-0022] Nagy, D. S. , A. Isaic , A. C. Motofelea , D. I. Popovici , R. G. Diaconescu , and S. M. Negru . 2024. “The Role of Spirituality and Religion in Improving Quality of Life and Coping Mechanisms in Cancer Patients.” Health 12: 2349. 10.3390/healthcare12232349 PMC11641253 39684971

[odi70335-bib-0023] Page, M. J. , J. E. McKenzie , P. M. Bossuyt , et al. 2021. “The PRISMA 2020 Statement: An Updated Guideline for Reporting Systematic Reviews.” BMJ 372: n71. 10.1136/bmj.n71. PMC8005924 33782057

[odi70335-bib-0024] Philipp, J. , S. S. Linsen , Z. Alexander‐Nicolai , et al. 2022. “Gender‐Specific Differences Concerning Psychosocial Aspects and Functional Impairments That Influence Quality of Life in Oral Cancer Treatment.” Supportive Care in Cancer 30, no. 6: 4905–4915. 10.1007/s00520-022-06907-9. 35171323

[odi70335-bib-0025] Popescu, B. , and O. Păun . 2017. “End‐Stage Head and Neck Cancer Coping Mechanisms.” Journal of Mind and Medical Sciences 4, no. 2: 93–99. 10.22543/7674.42.p9399.

[odi70335-bib-0026] Reis, L. B. M. , C. R. Leles , and M. C. M. Freire . 2020. “Religiosity, Spirituality, and the Quality of Life of Patients With Sequelae of Head and Neck Cancer.” Oral Diseases 26, no. 4: 838–842. 10.1111/odi.13284. 31957109

[odi70335-bib-0027] Reis, L. B. M. , C. R. Leles , and M. C. M. Freire . 2021. “Associations of Religiosity and Spiritual Well‐Being With Appearance Concerns After Head and Neck Cancer Surgery.” Community Dentistry and Oral Epidemiology 49, no. 5: 420–426. 10.1111/cdoe.12615. 33372313

[odi70335-bib-0028] Ripamonti, C. , G. Miccinesi , M. A. Pessi , P. Di Pede , and M. Ferrari . 2016. “Is It Possible to Encourage Hope in Non‐Advanced Cancer Patients? We Must Try.” Annals of Oncology 27, no. 3: 513–519. 10.1093/annonc/mdv614. 26681679

[odi70335-bib-0029] Santana, J. J. R. A. , C. R. Zanin , and J. V. Maniglia . 2008. “Pacientes com Câncer: Enfrentamento, Rede Social e Apoio Social.” Paidéia (Ribeirão Preto) 18, no. 40: 371–384. 10.1590/S0103-863X2008000200013.

[odi70335-bib-0030] Wallace, H. M. 2013. Getting to the Other Side: An Exploration of the Head and Neck Cancer Treatment Experience [Dissertation]. Walden University.

[odi70335-bib-0031] Wallace, H. M. 2019. “The Role of Spirituality and 12 Step Groups in Addressing Treatment Fear and Worry Among Head and Neck Cancer Patients.” Online Journal of Complementary & Alternative Medicine 2, no. 3: 10–14. 10.33552/ojcam.2019.02.000537.

[odi70335-bib-0032] Wenham, J. , M. Best , and D. W. Kissane . 2021. “Systematic Review of Medical Education on Spirituality.” Internal Medicine Journal 51: 1781–1790. 10.1111/imj.15421 34142417

[odi70335-bib-0033] Whitford, H. S. , and I. N. Olver . 2012. “The Multidimensionality of Spiritual Wellbeing: Peace, Meaning, and Faith and Their Association With Quality of Life and Coping in Oncology.” Psycho‐Oncology 21, no. 6: 602–610. 10.1002/pon.1937. 21370313

[odi70335-bib-0034] Wise, M. , L. R. Marchand , L. J. Roberts , and M. Y. Chih . 2018. “Suffering in Advanced Cancer: A Randomized Control Trial of a Narrative Intervention.” Journal of Palliative Medicine 21, no. 2: 200–207. 10.1089/jpm.2017.0007. PMC5797325 29135330

[odi70335-bib-0035] Wong, Y. K. , W. C. Tsai , J. C. Lin , et al. 2006. “Socio‐Demographic Factors in the Prognosis of Oral Cancer Patients.” Oral Oncology 42, no. 9: 893–906. 10.1016/j.oraloncology.2005.12.007. 16730220

[odi70335-bib-0036] Zhu, C. , Q. Chen , W. Du , H. Xu , and X. He . 2024. “Evaluating Psychological Distress in Chinese Patients With Head and Neck Squamous Cell Carcinoma Planned for Radiotherapy: A Cross‐Sectional Study Using the Distress Thermometer.” BMC Psychol 12, no. 1: 577. 10.1186/s40359-024-02081-1. PMC11490125 39427226

