# Supplementary material for: Spirituality and Religiosity in Adults With Head and Neck Cancer: A Scoping Review of Concepts, Measures, and Outcomes
Source: Oral Dis. 2026 Apr 14;32(8):2194–205. doi: 10.1111/odi.70335 (PMC13693625; doi:10.1111/odi.70335)
Supplement: Supplementary file 1 [file ODI-32-2194-s001.docx]

**Table 1S-** Preliminary search strategy.

| **Database** | **Query (December 16, 2024)** | **Results** |
| --- | --- | --- |
| PubMed/  MEDLINE | ("Head and neck cancer"[All Fields] OR "Oropharyngeal cancer"[All Fields] OR "Oral cancer"[All Fields] OR "Laryngeal cancer"[All Fields] OR "Nasopharyngeal cancer"[All Fields] OR "Hypopharyngeal cancer"[All Fields] OR "squamous cell carcinoma"[All Fields]) AND ("Spirituality"[All Fields] OR "Religiosity"[All Fields] OR "Spiritual care"[All Fields] OR "Spiritual well-being"[All Fields] OR "Pastoral care"[All Fields] OR "Religious support"[All Fields] OR "Faith-based care"[All Fields] OR "Spiritual counseling"[All Fields] OR "Holistic care"[All Fields] OR "End-of-life care"[All Fields] OR "Religious belief"[All Fields] OR "Spiritualism" OR "Spiritualities" OR "Pastoral Psychology") | 107 |
| Embase | ('head and neck cancer':ti,ab,kw OR 'oropharyngeal cancer':ti,ab,kw OR 'oral cancer':ti,ab,kw OR 'laryngeal cancer':ti,ab,kw OR 'nasopharyngeal cancer':ti,ab,kw OR 'hypopharyngeal cancer':ti,ab,kw OR 'squamous cell carcinoma':ti,ab,kw OR 'head and neck neoplasm*':ti,ab,kw OR 'oropharyngeal neoplasm*':ti,ab,kw OR 'oral neoplasm*':ti,ab,kw OR 'laryngeal neoplasm*':ti,ab,kw OR 'nasopharyngeal neoplasm*':ti,ab,kw OR 'hypopharyngeal neoplasm*':ti,ab,kw) AND ('spirituality':ti,ab,kw OR 'religiosity':ti,ab,kw OR 'spiritual care':ti,ab,kw OR 'spiritual well-being':ti,ab,kw OR 'pastoral care':ti,ab,kw OR 'religious support':ti,ab,kw OR 'faith-based care':ti,ab,kw OR 'spiritual counseling':ti,ab,kw OR 'holistic care':ti,ab,kw OR 'end-of-life care':ti,ab,kw OR 'religious belief':ti,ab,kw OR 'spiritualism':ti,ab,kw OR 'spiritualities':ti,ab,kw OR 'pastoral psychology':ti,ab,kw) | 144 |
| Lilacs | (("Câncer de cabeça e pescoço" OR "Cáncer de cabeza y cuello") OR  ("Câncer orofaríngeo" OR "Cáncer orofaríngeo") OR  ("Câncer oral" OR "Cáncer oral") OR  ("Câncer laríngeo" OR "Cáncer laríngeo") OR  ("Câncer nasofaringeano" OR "Cáncer nasofaríngeo") OR  ("Câncer hipofaringeano" OR "Cáncer hipofaríngeo") OR  ("Carcinoma de células escamosas" OR "Carcinoma de células escamosas")) E (("Espiritualidade" OR "Espiritualidad") OR  ("Religiosidade" OR "Religiosidad") OR  ("Cuidados espirituais" OR "Atención espiritual") OR  ("Bem-estar espiritual" OR "Bienestar espiritual") OR  ("Cuidados pastorais" OR "Atención pastoral") OR  ("Apoio religioso" OR "Apoyo religioso") OR  ("Cuidados baseados na fé" OR "Atención basada en la fe") OR  ("Aconselhamento espiritual" OR "Consejería espiritual") OR  ("Cuidados holísticos" OR "Atención holística") OR  ("Cuidados no fim da vida" OR "Atención al final de la vida") OR  ("Crença religiosa" OR "Creencia religiosa") OR  ("Espiritualismo" OR "Espiritualismo") OR  ("Espiritualidades" OR "Espiritualidades") OR  ("Psicologia pastoral" OR "Psicología pastoral")) | 8 |
| scielo | (("câncer de cabeça e pescoço" OR "cáncer de cabeza y cuello" OR "head and neck cancer") ) AND (("espiritualidade" OR "espiritualidad" OR "spirituality" OR "religiosidade" OR "religiosidad" OR "religiosity")) | 0 |
| scopus | TITLE-ABS-KEY(("head and neck cancer" OR "oropharyngeal cancer" OR "oral cancer" OR "laryngeal cancer" OR "nasopharyngeal cancer" OR "hypopharyngeal cancer" OR "squamous cell carcinoma")) AND TITLE-ABS-KEY(("spirituality" OR "religiosity" OR "spiritual care" OR "spiritual well-being" OR "pastoral care" OR "religious support" OR "faith-based care" OR "spiritual counseling" OR "holistic care" OR "end-of-life care" OR "religious belief" OR "spiritualism" OR "spiritualities" OR "pastoral psychology")) | 251 |
| web of science | TS=("head and neck cancer" OR "oropharyngeal cancer" OR "oral cancer" OR "laryngeal cancer" OR  "nasopharyngeal cancer" OR "hypopharyngeal cancer" OR "squamous cell carcinoma") AND TS=("spirituality" OR "religiosity" OR "spiritual care" OR "spiritual well-being" OR "pastoral care" OR "religious support" OR "faith-based care" OR "spiritual counseling" OR "holistic care" OR "end-of-life care" OR "religious belief" OR "spiritualism" OR "spiritualities" OR "pastoral psychology") | 94 |
|  | **Query (February 21, 2025)** |  |
| Google Scholar | "Head and neck cancer" OR "Oral cancer" OR "squamous cell carcinoma" AND "Spirituality" OR "Religiosity" OR "Spiritual care" OR "Spiritual well-being" OR "Pastoral care" | 100 |
| Proquest | TI,AB("Head and neck cancer" OR "Oropharyngeal cancer" OR "Oral cancer" OR "Laryngeal cancer" OR "Nasopharyngeal cancer" OR "Hypopharyngeal cancer" OR "squamous cell carcinoma") AND TI,AB("Spirituality" OR "Religiosity" OR "Spiritual care" OR "Spiritual well-being" OR "Pastoral care" OR "Religious support" OR "Faith-based care" OR "Spiritual counseling" OR "Holistic care" OR "End-of-life care" OR "Religious belief" OR "Spiritualism" OR "Spiritualities" OR "Pastoral Psychology") | 66 |
