# Supplementary material for: Spirituality and Religiosity in Adults With Head and Neck Cancer: A Scoping Review of Concepts, Measures, and Outcomes
Source: Oral Dis. 2026 Apr 14;32(8):2194–205. doi: 10.1111/odi.70335 (PMC13693625; doi:10.1111/odi.70335)
Supplement: Supplementary file 2 [file ODI-32-2194-s002.docx]

**Table 2S** - Measurement Instruments.

| **Category** | **Instrument** | **n** |
| --- | --- | --- |
| **Psychological Assessment** | Distress Thermometer (DT) | 2.0 |
|  | DAS24 – *Derriford Appearance Scale – 24 items* | 1.0 |
|  | Demoralization Scale | 1.0 |
|  | DS – *Depressive Scale* | 1.0 |
|  | HADS – *Hospital Anxiety and Depression Scale* | 1.0 |
|  | BDI – *Beck Depression Inventory* | 1.0 |
|  | BDA – *Anxiety Assessment (Portuguese)* | 1.0 |
|  | STAI – *State–Trait Anxiety Inventory* | 1.0 |
| **Quality of Life (QoL)** | FACT-H&N / FACT-HN – *Functional Assessment of Cancer Therapy – Head and Neck* | 2.0 |
|  | EORTC (QLQ-H&N35 module) | 1.0 |
|  | EORTC QLQ – *Taiwan Chinese Version* | 1.0 |
|  | HRQoL – *Health-Related Quality of Life* | 1.0 |
|  | QoL_CS – *Quality of Life – Cancer Survivor* | 1.0 |
|  | SF-12 – *Short Form 12 Health Survey Questionnaire* | 1.0 |
|  | UW-QOL – *University of Washington Quality of Life Questionnaire* | 1.0 |
|  | WHOQOL-Bref – *World Health Organization Quality of Life – Bref version* | 1.0 |
| **Spirituality / Religiosity** | DUREL – *Duke University Religiosity Index / Religiousness Index* | 2.0 |
|  | FACIT-Sp12 – *Functional Assessment of Chronic Illness Therapy – Spiritual Well-Being Scale* | 2.0 |
|  | ESAS-r-CSS – *Edmonton Symptom Assessment Scale – Revised (Constipation and Spiritual Symptoms)* | 1.0 |
|  | Modified Chinese Spiritual Needs Tool (21-item, Taiwan) | 1.0 |
|  | WHOQOL-SRPB – *WHOQOL Spirituality, Religiousness and Personal Beliefs* | 1.0 |
|  | The You, Your Family and Friends Scale | 1.0 |
|  | Measures of Social Networks and Support | 1.0 |
|  | F-SozU – *Questionnaire on Social Support* | 1.0 |
|  | CNAT – *Comprehensive Needs Assessment Tool in Cancer for Patients* | 1.0 |
|  | Bochum Questionnaire on Rehabilitation | 1.0 |
|  | WHQ – *Ways of Helping Questionnaire* | 1.0 |
| **Coping and Illness Beliefs** | Ways of Coping with Problems Scale | 1.0 |
|  | FKV – *Freiburg Questionnaire of Coping with Illness* | 1.0 |
|  | KKG – *Questionnaire on Control Convictions Regarding Illness and Health* | 1.0 |
